# Fatty Acid Composition of Fixed Oil of *Linum strictum* and Its Antibiofilm, Anticoagulant, and α‐Amylase Inhibitory Effects–In Vitro and In Silico Study

**DOI:** 10.1002/cbdv.71743

**Published:** 2026-09-20

**Authors:** Hayet Edziri, Pooja Bargali, Marwa Melliti, Hasan Aljohi, Mohammad NasserAlkhrayef, Rim Gtari, Marcello Iriti, Ravendra Kumar, Ibrahim O. Alanazi

**Affiliations:** ^1^ Laboratory of Transmissible Diseases and Biologically Active Substances LR99ES27, Faculty of Pharmacy University of Monastir Monastir Tunisia; ^2^ Department of Chemistry, College of Basic Sciences and Humanities G.B. Pant University of Agriculture and Technology U.S. Nagar Uttarakhand India; ^3^ Applied Genetics Technology Institute King Abdulaziz City for Science and Technology Riyadh Saudi Arabia; ^4^ Disability Research Institute King Abdulaziz City For Science and Technology Riyadh Saudi Arabia; ^5^ Department of Biomedical Surgical and Dental Sciences University of Milan Milan Italy; ^6^ The Healthy Aging Research Institute King Abdulaziz City For Science and Technology Riyadh Saudi Arabia

**Keywords:** antibacterial, antibiofilm, anticoagulation, *Linum strictum*, molecular docking, multi‐resistant bacteria, seed oil, α‐amylase

## Abstract

The present study investigated the fatty acid composition of *Linum strictum* seed oil and evaluated its antibacterial, antibiofilm, anticoagulant, and α‐amylase inhibitory activities. The oil was extracted from commercially obtained seeds and characterized by GC‐FID. Antibacterial activity was determined using the microbroth dilution method, while antibiofilm activity was assessed against multidrug‐resistant strains, including *Pseudomonas aeruginosa*, methicillin‐resistant *Staphylococcus aureus* (MRSA), and *Acinetobacter baumannii*. Anticoagulant effects were evaluated using prothrombin time (PT) and activated partial thromboplastin time (aPTT), and α‐amylase inhibition was assessed in vitro. The oil was characterized by a high proportion of unsaturated fatty acids, mainly α‐linolenic (57.08%), oleic (17.53%), and linoleic (14.0%) acids. Significant antibacterial activity was observed against *Enterococcus faecalis* ATCC 29212 and *A. baumannii* A2553 and A722 (MIC = 1.25 mg/mL), while three *S. aureus* strains showed MIC values of 5 mg/mL. Biofilm inhibition exceeded 70% against *A. baumannii* at 2×MIC. The oil prolonged coagulation times, particularly through the intrinsic pathway (PT = 16.4 s; aPTT = 81.6 s). α‐Amylase inhibition reached 85.98% (IC_50_ = 0.0134 mg/mL), comparable to acarbose. Molecular docking revealed favorable binding of α‐oleic acid to BEL‐1 β‐lactamase and α‐linolenic acid to DNA gyrase B.

## Graphical abstract

Chemical composition, in vitro biological activity and in silico molecular docking of fixed oil of *Linum strictum*.

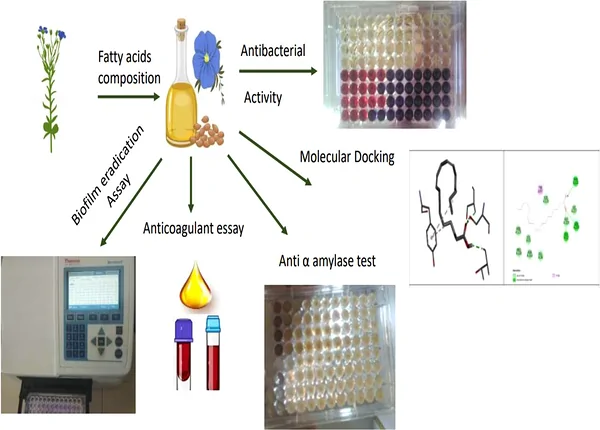

## 1 Introduction

*Linum* strictum, an annual herb from the genus *Linum* of the family *Linaceae*. *Linum* seed fixed oil is known as the primordial oil applied in varnishes, bio‐insecticides, paints, and herbicides. It contains flavonoids, phenols, and lignins, which can influence cell growth and survival [1]. The complex blend of bioactive elements found in *L. strictum* fixed oil includes minor chemicals like phytosterols, tocopherols, and other molecules derived from lipids, as well as unsaturated fatty acids like oleic, linoleic, and α‐linolenic acids [2].

It is one of the oldest commercial oils, and solvent‐processed flaxseed oil has been used for centuries as a drying oil in painting and varnishing. The raw oil is used as an astringent in fungicidal lotion [3]. The oil studied previously in Romania contains unsaturated fatty acids like oleic acid (18.51%) and linoleic acid (17.25%) [4]. These fatty acids appear to give the oil a drying property. In a preliminary study, *L. strictum* fixed oil has been found to inhibit inflammation [5]. The therapeutic effect of *L*. strictum fixed oil on acute and chronic arthritic albino rat models has been reported. The antiulcer activity of *L*. strictum fixed oil in animal models has been previously demonstrated [6].

The fixed oil of the wild *Linum* species *L. strictum* has a high percentage of unsaturated fatty acids. According to earlier research, the main components are α‐linolenic acid (ALA), linoleic acid (LA), and oleic acid (OA), albeit their relative abundance can change based on genetics and environmental factors. The main polyunsaturated fatty acids in *L. strictum* are typically α‐linolenic acid and linoleic acid, while oleic acid is found in smaller amounts, though their relative amounts may differ depending on the species, geographic origin, environmental factors, and extraction techniques. The oil's strong nutritional value and biological potential are attributed to this fatty acid composition [7].

In fact, unsaturated fatty acids have been shown to have antibacterial properties as well as to prevent the formation of bacterial biofilms by interfering with bacterial adhesion, quorum sensing, and membrane integrity. Thus, *L. strictum* fixed oil's antibacterial activities may be influenced by its fatty acid content [8].

In this context, oleic acid has been linked to cardioprotective, antioxidant, and anti‐inflammatory properties. A vital omega‐6 fatty acid, linoleic acid, has been shown to have antibacterial and wound‐healing qualities and is crucial for preserving the integrity of cell membranes. The antioxidant, anti‐inflammatory, hypoglycemic, anticoagulant, and cardiovascular health benefits of α‐linolenic acid, an important omega‐3 fatty acid, have garnered significant attention [9].

Therefore, considering the importance of this oil in the human diet and for human health, the objective of the present study was to evaluate the fatty acid composition, the antibiofilm effect toward multidrug‐resistant bacteria, and finally, the anticoagulant potential of *L. strictum* fixed oil. Molecular docking has become an important complementary tool in natural product research because it predicts ligand–protein interactions, provides mechanistic insights into the observed biological activities, and supports the interpretation of experimental findings. Accordingly, in the present study, molecular docking was performed as a complementary computational approach to explore plausible interactions between the identified bioactive compounds and biologically relevant target proteins rather than to establish direct evidence of ligand binding or mechanism of action.

## 2 Materials and Methods

### 2.1 Plant Material

*Linum* strictum seeds were purchased at a local market in Jeddah (Arabie saudie) in May 2025, and the voucher specimens (voucher number LU1212) were delivered to our lab. The identification of the plant was assessed by Prof. Fethia Skhiri, a botanist at the Monastir High Institute of Biotechnology in Tunisia.

### 2.2 Extraction of Fixed Oil of *L. strictum*

The fixed oil used in this study was extracted in our laboratory from commercially purchased seeds.

For oil extraction for 7 days, pulverized seeds were cold‐macerated in petroleum ether at 40°C–60°C. After the petroleum ether was removed from the sample, the oil was filtered until it was clear. The oil was kept in an amber‐colored, sealed bottle at room temperature. The oil was loaded to the brim of the bottle, leaving no headspace, and then purged with nitrogen to prevent oxidation. Regarding dried seeds, the yield of fixed oil was 17.50% v/w. The oil had a density of 0.952 g/mL.

### 2.3 Fatty Acid Composition of *L. strictum* Fixed Oil by GC‐FID

Gas–liquid chromatography with flame ionization detection (GC‐FID) was used to measure fatty acids following the crude fat extraction and derivatization processes outlined by [10]. A DANI model GC 1000 apparatus with a Macherey‐Nagel column, a FID, and a split/splitless injector was used to perform the analysis. In short, methanol containing sulfuric acid was added to the oil sample, heated, and then extracted using n‐hexane to create fatty acid methyl esters (FAMEs). A capillary column appropriate for FAME analysis (100 m × 0.25 mm i.d. × 0.20 µm film thickness) was used for separation. The carrier gas was helium at a constant flow rate of 1.0 mL/min. An injection volume of 1 µL was used, with a split ratio of 10:1. The temperatures of the injector and detector were kept at 250°C and 260°C, respectively.

The oven temperature program was as follows: initial temperature was 80°C held for 2 min, increased at 15°C/min to 168°C, and held for 18 min. Finally, it was increased to 186°C at 5°C/min and maintained for 23 min. Fatty acids were identified by comparing their retention times with those of a commercial FAME standard (Sigma‐Aldrich). Peak identification was confirmed by retention times of individual FAME standards. The results were presented as relative percentages of total fatty acids. The Clarity DataApex 4.0 software was used to record and process the results, which were then expressed as a relative percentage of each fatty acid.

### 2.4 Assessment of Biological Properties

#### 2.4.1 Determination of Minimal Inhibitory Concentration of Fixed Oil

Several common and resistant clinically isolated bacterial strains from the microbiology lab at the hospital of Fattouma Bourguiba Monastir were used for antimicrobial tests.

As previously mentioned, the microdilution method was used to acquire the MIC values [11]. The fixed oil was tested at concentrations ranging from 0.625 to 20 mg/mL. Twenty microliters of standardized bacterial cell suspension (McFarland 0.5)

Wells containing 100 µL of BH broth were filled with (1.5 CFU mL^−1^). At 37°C, the microplates were incubated for 24 h.

The 10% DMSO solvent used to dissolve the oil was included as a negative control at the same final concentration used in the assays. Ciprofloxacin was used as the positive control for antibacterial assays.

The final tube that showed no bacterial growth after incubation was chosen to represent the least inhibitory concentration (MIC).

The minimal bactericidal concentration (MBC) was the lowest concentration from which the bacteria did not recover when the sample exhibiting no turbidity was subcultured. Both positive and negative control samples were incubated in the same manner. Additionally, MBC/MIC ratios were computed. If this ratio is larger than 4, the sample is regarded as bacteriostatic; if it is less than or equal to 4, it is regarded as bactericidal [12].

#### 2.4.2 Biofilm Eradication of Fixed Oil

Only *S. aureus* (MRSA), *Pseudomonas aeruginosa*, and *Acinetobacter baumannii* bacteria were investigated for biofilm formation eradication, with final concentrations matching MIC and 2xMIC. With only slight adjustments, the methodology of a prior study was used [11].

Suspensions from an overnight bacterial culture (final concentration of CFU/mL) were made in a Thio tube with 2% glucose. A 96‐well microplate was then filled with 50 µL of the bacterial culture, 100 µL of the sample with the final concentration, and 50 µL of Thio. As positive and negative controls, the test included wells that had only bacteria and wells that did not contain any other materials. The contents of every well were aspirated following an overnight culture at 37°C. Following two rounds of distilled water washing to get rid of non‐adherent bacteria, wells were fixed with 200 µL of 95% ethanol.

Following 15 min, 100 µL of 0.1% crystal violet was added and left for 5 min. After removing the crystal violet, distilled water was used to wash the plate three times. Triplicates of each experiment were conducted. The absorbance of the bacterial growth without extract, or ODc (growth control), and the absorbance of the extract with bacteria, or ODs (growth sample), were measured at 590 nm.

The following formula was used to determine the percentage of biofilm eradication:

The percentage of inhibition of biofilms:

$$\begin{array}{ccc} & & \mathrm{Biofilm}\;\mathrm{eradication}\;\mathrm{percentage}\\ & & \;=\left(\frac{\mathrm{OD}\;\mathrm{control}\;-\;\mathrm{OD}\;\mathrm{sample}}{\mathrm{OD}\;\mathrm{control}}\;\right)\;\times 100 \end{array}$$

### 2.5 Anticoagulant Activity

#### 2.5.1 Prothrombin Time (PT) Test

50 µL of pooled plasma and 1 µL of the sample dissolved in 10% DMSO or heparin (positive control) were combined, and the mixture was incubated for 1 min at 37°C. Calcium thromboplastin (200 µL) was then added. A digital coagulometer was used to record the clotting time. Different concentrations of the oil were examined for the tests [1].

#### 2.5.2 Kaolin Clotting Time (KCT)

After adding a material that triggered the intrinsic system and accessed the functionality of factors engaged in this pathway, the kaolin clotting time test (KCT) assesses the ability of plant extracts to clot blood. With a few adjustments, the activity of the oil was assessed [11]. 50 µL of pooled plasma, 1 µL of each sample diluted in 10% DMSO, and 50 µL of the activator “C.K PREST” were combined, and the mixture was incubated for 3 min at 37°C. Next, 50 µL of 0.025 M calcium chloride that had been heated to 37°C and 100 µL of cephalin kaolin were added. A digital coagulometer was used to record the clotting time. Different concentrations (5, 10, 20, and 40 mg/mL) were examined in the studies. Three separate evaluations of the anticoagulation assay were conducted. All experiments were performed in triplicate and repeated independently three times.

### 2.6 α‐Amylase Inhibition Activity

With some adjustments, the α‐amylase inhibition test was carried out in triplicate using the approach previously outlined [13]. The studied oil utilized had concentrations ranging from 0.009 to 1.25 mg/mL.

50 microliters of the α‐amylase solution (0.5 mg/mL) with 0.02 M sodium phosphate buffer (pH 6.9 with 0.006 M NaCl) and 10 microliters of the oil were mixed in a 96‐well plate, and the mixture was incubated for 10 min. 100 µL of a starch (1%) solution in 0.02 M sodium phosphate buffer (pH 6.9 with 0.006 M sodium chloride) was added following the pre‐incubation phase, and the mixture was then incubated for 3 min at 25°C.

125 µL of a 3,5‐dinitrosalicylic acid reagent was used to complete the reaction. It was then cooled to room temperature, diluted with 200 µL of deionized water, and placed in a boiling water bath for 5 min. At 540 nm, the absorbance was then measured.

Acarbose was employed as the positive control at the same concentration as the oil, and the absorbances of the control samples, which were made using a buffer solution instead of the extract and amylase—were also measured. All experiments were performed in triplicate and repeated independently three times. The IC_50_ values were determined using GraphPad Prism 7.0 by nonlinear regression analysis of the concentration–response curves using a four‐parameter logistic model. The following formula was used to calculate the amylase inhibition:

$$\%\;\alpha -\mathrm{Amylase}\;\mathrm{Inhibition}\;=\left(\frac{\mathrm{OD}\;\mathrm{control}\;-\;\mathrm{OD}\;\mathrm{sample}}{\mathrm{OD}\;\mathrm{control}}\;\right)\;\times 100$$

where: OD control: The untreated sample's optical density.

OD sample: The treated sample's optical density.

### 2.7 Molecular Docking

**FIGURE 1:**
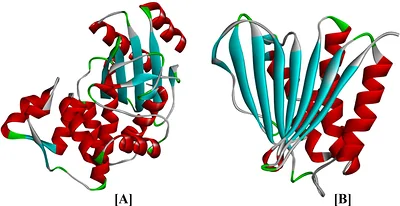
3D structures of bacterial target proteins (a)  β‐lactamase BEL‐1 (PDB ID :5EUA); (b)  DNA gyrase B (PDB ID : 4URO).

Molecular docking studies were performed to evaluate interactions between major compounds from *L. strictum* seed oil and two bacterial target proteins, β‐lactamase BEL‐1, and DNA gyrase B. These target proteins were used for docking studies because they have been reported in previous antibacterial studies. Bacterial DNA gyrase is an essential enzyme involved in DNA replication, transcription, and recombination, making it a key antibacterial target. Additionally, *P. aeruginosa* expressing the BEL‐1 extended‐spectrum β‐lactamase hydrolyses a broad range of β‐lactam antibiotics, including carbapenems, thereby contributing to multidrug resistance and posing a significant clinical challenge. Docking simulations were conducted using PyRx software with the AutoDock Vina Wizard. The three‐dimensional structures of β‐lactamase BEL‐1 (PDB ID: 5EUA) and DNA gyrase B (PDB ID: 4URO) were retrieved from the RCSB Protein Data Bank. Protein structures were prepared in Biovia Discovery Studio 2021 by removing water molecules and HETATOMS and adding polar hydrogens. Major oil constituents: palmitic acid, oleic acid, linoleic acid, α‐linolenic acid, and stearic acid were selected for docking based on their relative abundance and evaluated against the standard compound, ciprofloxacin (used as a standard in antibacterial activity). Ligand structures were obtained from the PubChem database in SDF format. Ligand preparation in PyRx included energy minimization using the universal force field (UFF), charge optimization, and conversion to the AutoDock‐compatible PDBQT format. Docking was executed using the Vina Wizard module, with binding affinities calculated for each ligand–protein complex. The resulting docking scores were exported as CSV files for further analysis. Visualization of 2D ligand–protein interactions and binding poses was performed in Biovia Discovery Studio 2021. Docking validation was performed by re‐docking the native co‐crystallized ligands Novobiocin and Moxalactam into the protein's 4URO and 5EUA active sites, respectively. The protocol was validated by comparing the re‐docked and original ligand poses, with RMSD values of 1.20 Å (4URO) and 1.98 Å (5EUA) confirming the accuracy and reliability of the docking method. The docking search space was defined using a grid box centered for 4URO at (X = −1.84, Y = −12.92, Z = −6.18) with dimensions of 40.23× 43.82 × 45.94 Å, and for 5EUA (X = −17.44, Y = −1.23, Z = −8.50) with dimensions of 49.28 × 50.37 × 47.85 Å, covering the entire active site to allow efficient ligand sampling. Figure 1 illustrates the 3D structures of the target proteins used for the docking studies.

### 2.8 Statistical Analysis

All experiments were performed in three independent experiments (*n* = 3), with each experiment conducted in triplicate. Data are expressed as mean ± standard deviation (SD). Statistical analyses were performed using GraphPad Prism version 7.0 (GraphPad Software, San Diego, CA, USA). The statistical difference between the samples was ascertained using an analysis of variance (ANOVA) and the Tukey test. Microsoft Excel was used to calculate the data's mean and standard deviations.

## 3 Results and Discussion

### 3.1 Fatty Acid Analysis of Fixed

**FIGURE 2:**
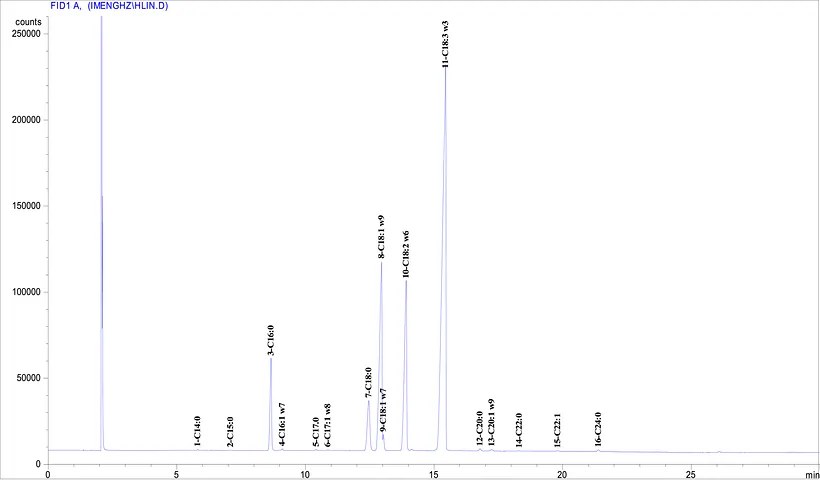
Chromatogram of *L. strictum* oil by GC/FID.

The chemical composition of *L. strictum* seed oil is presented in Table 1 and Figure 2.

**TABLE 1:** Chemical composition of *L. strictum* seed fixed oil by GC/FID.

Types of fatty acids	Names of fatty acids	Formulas	Lin seed oil (%)
Saturated fatty acids	Myristic acid	C14:0	0.07
	Pentadecylic acid	C15:0	0.05
	Palmitic acid	C16:0	4.57
	Margaric acid	C17:0	0.10
	Stearic acid	C18:0	4.87
	Arachidic acid	C20:0	0.18
	Behenic acid	C22:0	0.14
	Lignoceric acid	C24:0	0.26
Total saturated fatty acids			10.24
Unsaturated fatty acids	Palmitoleic acid	C16:1 w7	0.08
	Vaccenic acid	C18:1 w7	0.66
	Linoleic acid	C18:2 w6	14.00
	α‐Linolenic acid	C18:3 w3	57.08
	Oleic acid	C18:1 w9	17.53
	Gadoleic acid	C20:1 w9	0.16
	Erucic acid	C22:1	0.15
Total unsaturated fatty acids			89.75
Total fatty acids			100.00

According to the data, *L. strictum* seed oil has a higher concentration of unsaturated fatty acids (89.75%), including linoleic acid (14%), oleic acid (17.53%), and alpha‐linolenic acid (57.08%). However, according to Blayo (2002) [14], it is lower in saturated fatty acids (10.24%), with stearic acid having the highest percentage (4.87%) and palmitic acid coming in second (4.57%).

Cultivated *L. strictum* seed oil includes 35% to 60% linolenic acid, 12% to 34% oleic acid, and 17% to 24% linoleic acid, according to a study done in 2002 by Blayo [14]. Variations in climatic, edaphic, geographical, and genotypic variables may be the cause of these variations in the chemical composition [15, 16].

Likewise, according to a recent study, 15 distinct fatty acids were identified in the seed oils of *L*. strictum, including palmitic, oleic, and linoleic acids as the predominant components [17].

In line with our research, Ghaleshahi et al. (2020) proposed that α‐linolenic acid was the most prevalent fatty acid, with oleic, palmitic, and stearic acids coming in second and third, respectively [18].

### 3.2 Antibacterial Potential

*L*. strictum fixed oil was tested for antibacterial property against multiple bacterial strains, including *S.aureus, E. coli, A.baumannii, P. aeruginosa* and, *E. faecalis* bacteria, results are gathered in Table 2.

**TABLE 2:** Antibacterial activity of *L. strictum* green oil.

Strains		IC (mg/mL)	MBC (mg/mL)	MIC/MBC	Ciprofloxacin MIC
Gram positive bacteria	*Enterococcus faecalis ATCC* 29212	1.25	5	4	0.125
	*S. aureus ATCC* 6583	5	10	2	0.031
	*S. aureus (MRSA) C 6960*	5	20	4	0.062
	*S. aureus (MRSA) A* 4620	10	20	2	0.062
	*S. aureus (MRSA) A* 3443	5	20	4	0.062
	*S. aureus (MRSA)A* 2953	10	20	2	0.062
	*S. aureus A(MRSA)*2861	10	20	2	0.062
Gram negative bacteria	*P. aeruginosa* ATCC	40	80	2	0.125
	*E. coli* ATCC	10	20	2	0.062
	*P. aeruginosa* A 861	20	20	1	0.125
	*P. aeruginosa* A 443	20	40	2	0.250
	*P. aeruginosa* A 862	20	40	2	0.250
	*P. aeruginosa* A 942	20	40	2	0.250
	*P. aeruginosa* A 2688	20	40	2	0.125
	*P. aeruginosa* A 2689	20	40	2	0.250
	*A. baumannii* A722	1.25	2.5	2	0.002
	*A. baumannii* B2049	2.5	2.5	1	0.002
	*A. baumannii* A2172	2.5	2.5	1	0.004
	*A. baumannii* A2553	1.25	2.5	2	0.004

Abbreviations: MRSA, methicillin‐resistant *S. aureus*; MIC, minimum inhibitory concentration; MBC, minimum bactericidal concentration (mg/mL); Ciprofloxacin, positive control.

Our findings showed that MIC values of *L*. strictum fixed oil ranged from 1.25 to 40 mg/mL. The best effect was pronounced against *Enterococcus faecalis ATCC* 29212*, A. baumannii* A722, *Acinetobacter baumannii* A2553 with MIC = 1.25 mg/mL. Furthermore, the MBC/MIC ratio was less than 4 for most of the tested bacteria; thus, the fixed oil exerted bactericidal activity [11].

A recent study on three Linseed oils had MIC and MBC values comparable to the employed positive controls (E211 and E224) and showed the strongest activity against the majority of the tested bacteria, particularly *Enterobacter cloacae* and *Escherichia coli* (MIC = 0.5,1 mg/mL). Similarly, the second linseed oil showed an antibacterial effect against *S.aureus* ATCC bacteria with MIC = 4 mg/mL [17].

A previous study carried out by Kaithwas et al. (2011) [18] reported that *L. strictum* fixed oil exhibited antibacterial efficacy against *S. agalactiae* and *S. aureus* equivalent to that of cefoperazone, but it was more effective against *E. faecalis, M. luteus, B. subtilis*, and *C. albicans*. With the exception of *B. pumilus*, which displayed a MIC of 0.3% (v/v) or 2.8 mg/mL, the MIC of fixed oil was found for all strains at a concentration of 0.2% (v/v) or 1.9 mg/mL (the weight/mL of oil is 0.952).

Furthermore, a disc diffusion method performed by Hussein and Azizi, (2021) [19], demonstrated that the alcoholic extract of *L. strictum* displayed an antibacterial action against *Salmonella typhimurium* (IZD = 19 mm) and *Shigella flexneri* (IZD = 20 mm).

A study elaborated previously in Jordan demonstrated that *Linum pubescens* essential oil had moderate antimicrobial activity against Gram‐negative bacteria (*E. coli* ATCC (MIC = 500 µg/mL), *and Klebsiella pneumoniae* ATCC (MIC = 600 µg/mL)) and high antimicrobial activity against Gram‐positive bacteria (*Bacillus cereus* ATCC (MIC = 500 µg/mL),), *Enterococcus faecalis* (MIC = 300 µg/mL), and *Staphylococcus aureus* ATCC (MIC = 150 µg/mL) [20].

According to Melliti et al. (2024) [21], the chemicals found in the oil could be responsible for this activity. The fatty acid content of the *L. strictum* seed oil, particularly the long‐chain ones with 12–18 carbons, may have contributed to the antibacterial qualities.

A previous study admitted that fatty acid production is inhibited, which is how unsaturated fatty acids work against bacteria [22].

According to earlier research, linoleic acid significantly blocked enoyl‐acyl carrier protein reductase (FabI), a crucial component of bacterial fatty acid production. The inhibition of (FabI) was similarly demonstrated by other unsaturated fatty acids, including oleic and linolenic acids [23].

Another study revealed that with minimum inhibitory concentrations (MIC) ranging from 0.01 to 1.0 mg/mL, linoleic acid reduced the development of every Gram‐positive bacterial species examined, including *Bacillus cereus, B. pumilus, B. subtilis, Micrococcus kristinae*, and *S. aureus* [24].

The high concentration of unsaturated fatty acids, especially oleic, linoleic, and α‐linolenic acids, in *L. strictum* fixed oil is probably responsible for its antibacterial properties. It is well known that these fatty acids interact with bacterial cell membranes, increasing membrane permeability, allowing intracellular components to seep out, and interfering with vital metabolic activities, all of which eventually impede bacterial development. The lack of an outer membrane, which makes it easier for hydrophobic substances to enter, may account for the increased vulnerability shown in Gram‐positive bacteria. Furthermore, rather than coming from a single ingredient, the oil's antibacterial activity might be the consequence of synergistic interactions between its various fatty acids and minor bioactive compounds [8].

This activity should not be attributed only to the main unsaturated fatty acids found in *L. strictum* fixed oil, even though they are likely to play a significant role in the biological activities that have been observed. Phytosterols, tocopherols, phenolic compounds, and other lipid‐derived molecules are examples of minor constituents found in complex combinations called fixed oils, which may also have biological action. Consequently, the combined effect of *L. strictum* fixed oil's chemical elements is more likely to produce its biological characteristics than any one of them alone. To determine the relative contributions of each component and their possible synergistic interactions, more research involving the fractionation of the oil and assessment of isolated chemicals will be required [25].

### 3.3 Biofilm Eradication of Fixed Oil

**FIGURE 3:**
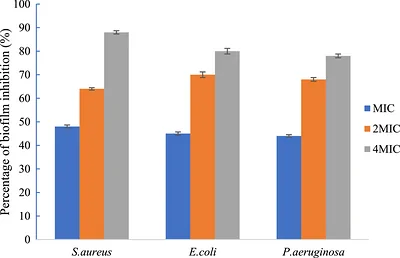
Antibiofilm activity of green oil of *L. strictum* against resistant bacteria.

The results of the antibiofilm effect displayed by *L. strictum* fixed oil are gathered in Figures 3 and 4.

FIGURE 42D binding interaction of ligands in the active site of the β‐lactamase BEL‐1 (PDB ID: 5EUA) (a) Palmitic Acid‐5EUA; (b) Oleic Acid‐5EUA; (c) Linoleic Acid‐5EUA; (d) α‐Linolenic Acid‐5EUA; (e) Stearic Acid‐5EUA; (f) Ciprofloxacin‐5EUA.

**Figure:**
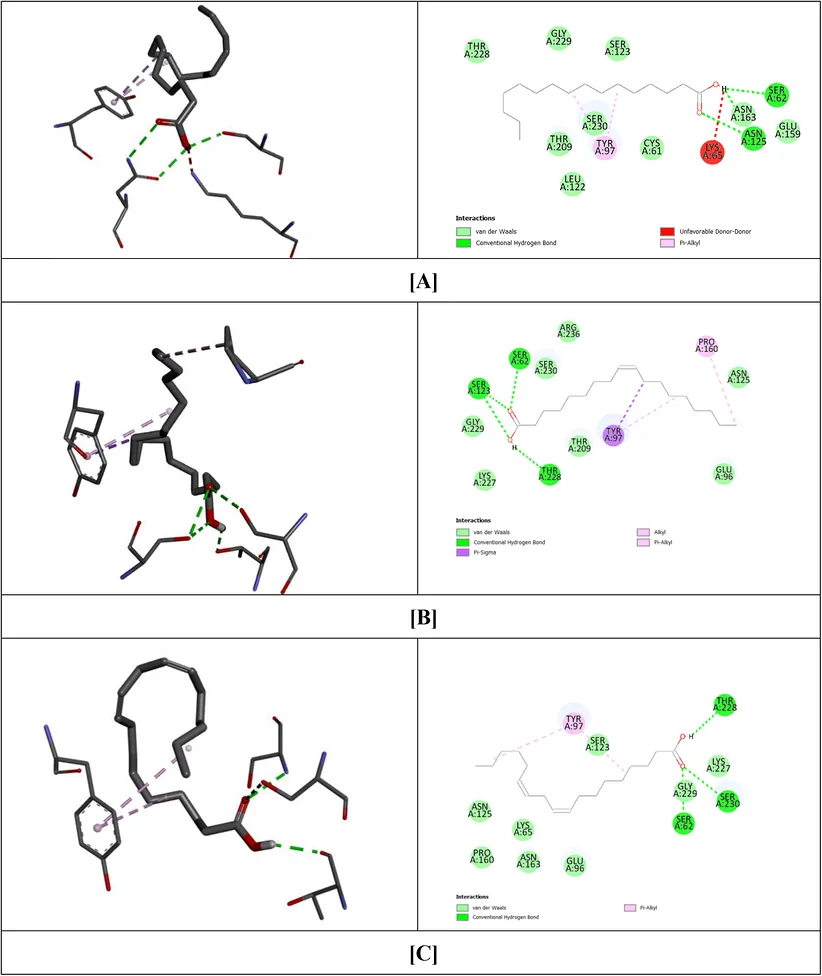

**Figure:**
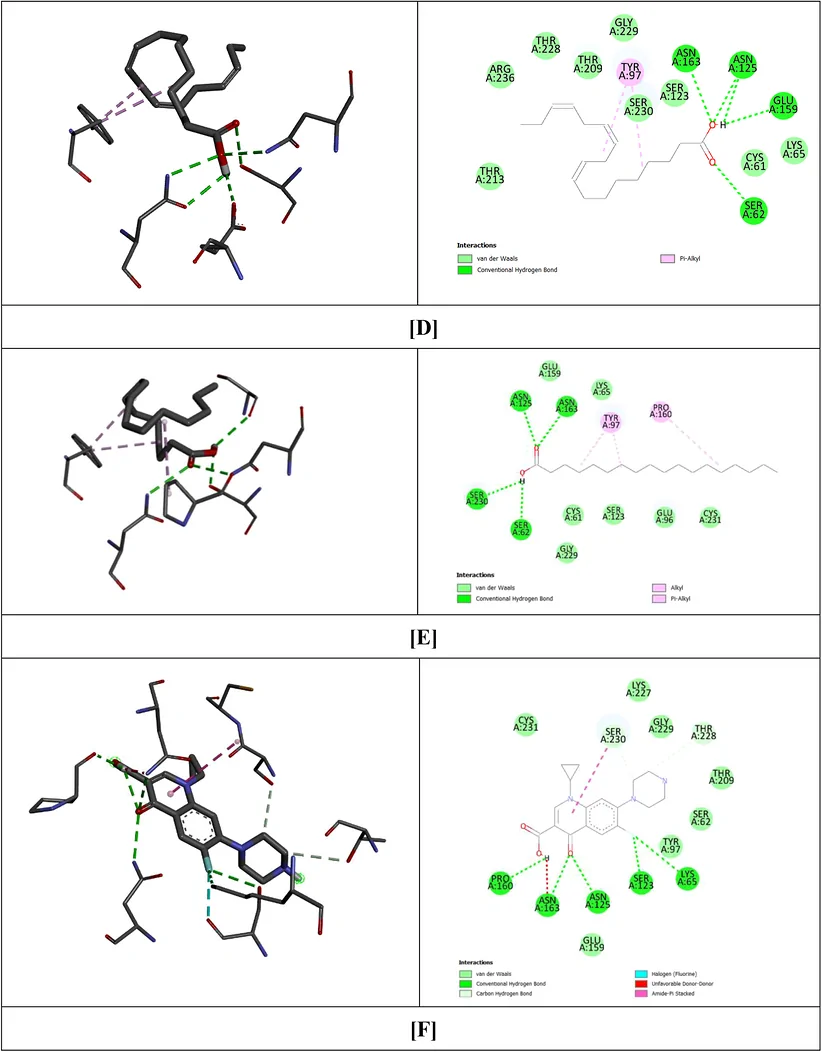

The fixed oil significantly inhibited biofilm formation in a dose‐dependent manner with (*p* < 0.05).

Because of their clinical significance, ability to form persistent biofilms, and correlation with antibiotic resistance, MRSA, *P. aeruginosa*, and *A. baumannii* were chosen as exemplary biofilm‐forming bacteria [26].

According to Sánchez et al. (2016) [27], biofilm inhibition is regarded as a key therapeutic target for the management of numerous bacterial illnesses. Multi drug resistance bacteria such as *S. aureus (*MRSA*), A.baumannii*, and *P.aeruginosa* were treated with green oil of *L*. strictum, and the results showed that the best activity was observed against *Acinetobacter* (70% at 2MIC), in addition, the inhibitory biofilm effect was obtained in a dose‐dependent manner.

As previously confirmed, the crude extract *of L*. strictum showed a great inhibitory capacity, especially against the biofilm formed by *S.aureus* bacteria at a concentration of 12.5 mg/mL [28].

Furthermore, flaxseed oil was tested for its antibiofilm effect against multi‐resistant bacteria, including MRSA, *S.epidermidis* bacteria, and a reduction of viable bacterial count was noticed as it measured by OD_490_ [29].

Numerous studies related the antibiofilm effect with the chemical composition of the plant extract [1, 30], in fact, methanolic extract of *Sapindus mukorossi* rich in oleic acid inhibited at a concentration of 250 µg/mL the formation of MRSA biofilm (82% of inhibition) [31].

One important virulence factor that increases bacterial persistence and resistance to antimicrobial treatments is biofilm development. Because *L. strictum* fixed oil contains unsaturated fatty acids that might obstruct bacterial adhesion in the early stages of biofilm development, it may have an inhibitory effect on biofilm formation. These substances have also been shown to change the fluidity of membranes and interfere with quorum‐sensing signaling pathways, which limits the development of biofilms and decreases bacterial communication. The oil may help prevent persistent biofilm‐associated infections, according to the demonstrated antibiofilm action [32].

In addition, numerous fatty acids, including linoleic and oleic acid, had an interesting antibiofilm potential (Yuyama et al., 2020). These acids according to an in silico study interfere with the quorum sensing homoserine lactone synthase enzyme, oleic acid displayed antibiofilm effect up to 57% against *A.baumanii* bacteria at low concentration (20 µg/mL) [33].

However, the potential synergistic interactions among the various components of the fixed oil should be further explored through fractionation and assessment of individual molecules, even though the primary fatty acids were thought to be significant contributors to the reported effects.

### 3.4 Anticoagulant Potential

The anticoagulant activity of the vegetable oil, was evaluated in vitro using the prothrombin time (PT) test, which explores the extrinsic pathway and the common pathway of blood coagulation, where tissue factor (thromboplastin) is the trigger for this pathway. On the other hand, the partial thromboplastin‐kaolin time (PTK) test was used, which explores the intrinsic pathway. When compared to the untreated control, the fixed oil treatment caused a statistically significant increase in clotting time (*p* < 0.05).

The clotting times obtained on normal plasma in the presence of oil were higher than the negative control, indicating the absence of anticoagulant activity on the exogenous pathway; however, anticoagulant activity on the endogenous coagulation pathway was noted.

The results presented in Table 3 revealed that *L. strictum* seed oil showed weak anticoagulant activity on the extrinsic pathway, with a 0.4 s prolongation of the TQ for a volume of 20 µL. On the other hand, a significant prolongation of 81.6 of the TCK for a volume of 40 µL compared to the control shows significant anticoagulant activity on the intrinsic pathway (Table 3).

**TABLE 3:** Anticoagulant activity of green oil of *L. strictum*.

Oil	Volume (µL)	PT	aPTT
*L. strictum*	**5**	14.1 ± 0.51^a^	56 ± 1.21^b^
	**10**	14.4 ± 1.03^a^	57.05 ± 2.11^b^
	**20**	15.3 ± 2.^42ab^	60.25 ± 2.67^b^
	**40**	16.4 ± 2.98^b^	81.6 ± 1.76^c^
Salin	**Salin**	14.9 ± 0.5^a^	33.7 ± 0.98^a^
Heparin	**1IU/mL**	40.8 ± 4.79^c^	111.8 ± 1.58^d^

Heparin, positive control; Salin, Negative control.

Data are expressed as the mean ± SD (*n* = 3). Differences between treatments (taken to the control) were determined using Tukey's post hoc test. Significant differences between treatment groups are represented by different letters, and equal letters indicate no significant difference between treatments. The results were expressed as mean ± SEM with *n* = 3.

The anticoagulant action of *L. strictum* fixed oil is in line with other studies that detailed the coagulation‐modulating characteristics of products derived from plants. *Cupressus sempervirens* extract showed strong anticoagulant activity in vitro, according to Al‐Rajhi et al. (2023) [34], indicating that bioactive phytochemicals found in plant matrices may disrupt the coagulation cascade. *Thevetia peruviana* latex's anticoagulant properties were also revealed by Al‐Rajhi et al. (2022) [35], underscoring the possible function of natural substances in controlling blood coagulation pathways. The anticoagulant impact of *L*. *strictum* fixed oil may be linked to its high concentration of unsaturated fatty acids, especially linoleic and α‐linolenic acids, which have been shown to affect hemostatic processes and platelet function. However, variations in chemical composition, particularly the kind and concentration of bioactive elements, may be linked to variations in anticoagulant potency among plant species. Therefore, the combined effects of *L. strictum* fixed oil's major fatty acids and small lipid components, which may work in concert to modify coagulation‐related pathways, may be the cause of its observed anticoagulant activity.

In fact, a recent study reported that phenolic extracts obtained from flaxseeds showed a moderate anti coagulation effect, methanolic extract displayed with a volume of 10 µL that KCT = 31.2 s and 29.5 s for the aqueous extract [36].

Another study demonstrated that flaxseed buffer extract demonstrated anticoagulant potential; the authors explained this activity by preventing ADP and epinephrine‐induced platelet aggregation. This extract demonstrated antiplatelet efficacy; the observed aggregation inhibition was 63.6% and 16.3%, respectively. Furthermore, it degraded all the human fibrin clot's chains as well as the Aα and Bβ chains of human fibrinogen [37].

In fact, fatty acids have been previously tested for their anti‐coagulation effects [38], such as oleanolic acid exhibited a potent anti coagulation capacity, it suppressed the production of either thrombin or FXa in human endothelial cells as well as the activity of both proteins. Additionally, it prevented human endothelial cells from expressing tissue factors that were activated by TNF‐α [39].

Furthermore, in mice with collagen‐adrenaline‐induced thrombosis, alpha‐linolenic acid and its combination with linoleic acid can increase the survival rate and lengthen the bleeding and coagulation times [40].

The high percentage of polyunsaturated fatty acids, especially α‐linolenic acid, may account for the anticoagulant action found in this study. According to earlier research, omega‐3 fatty acids can prolong clotting times by reducing platelet aggregation and modifying many coagulation cascade phases. Additionally, by affecting membrane lipid composition and eicosanoid synthesis, these fatty acids may have an impact on thrombin production and platelet activation. The current research supports the relevance of the fatty acid profile to the anticoagulant potential of *L. strictum* fixed oil, even though the exact mechanism is yet unknown [41].

### 3.5 α‐Amylase Inhibition Activity

The results of the anti‐α‐amylase effect are illustrated in Table 4 below. The α‐amylase inhibitory activity of the fixed oil was slightly higher than that of the positive control, with no statistical significance. Our findings revealed an interesting inhibitory potential of *L. strictum* seed oil, which showed 85.98% of inhibition with an IC_50_ value of 0.0134 mg/mL compared with acarbose as the positive control showing 84.93% of inhibition and the measured IC_50_ was 0.015 mg/mL.

**TABLE 4:** α‐amylase inhibition activities in *L. strictum* at a concentration of 1.25 mg/mL.

	Oil	Acarbose
Percentage of inhibition	85.98^a^ ± 0.12	84.93^a^ ± 0.08
IC_50_ (mg/mL)	0.0134^a^ ± 0.051	0.015^a^ ± 0.0001

Results are shown as mean  ± SD (*n* = 3) when compared to acarbose as a positive control. According to Tukey's test, means that are followed by the same letter do not differ significantly (*p* < 0.05).

A disadvantage of the current spectrophotometric assay is that possible effects related to dispersion and/or optical interference cannot be totally ruled out because the fixed oil is a lipid‐rich sample.

Our findings showed a better α‐amylase inhibitory effect compared with what had been previously reported by Al Qahtani et al. (2022), announcing that *L. strictum* ethanolic seed extract displayed a moderate inhibition potential of α‐ glucosidase enzyme activity (31.25% at 10 mg/mL) and the measured IC_50_ of α‐amylase was 8.54 mg/mL [42].

Mechchate et al. (2021) [43] showed that polyphenols extracted from *L. strictum* (PLU) treatment significantly improved the FBG blood glucose levels of diabetic mice.

Furthermore, after 28 days administration of aqueous extract of *L. strictum* seeds, a significant decrease in blood glucose was found in streptozotocin‐induced diabetic rats [44].

It was found previously that lignans from *L. strictum* showed a strong pancreatic amylase inhibitory effects and explained that action by the interaction of lignans with the active site of the enzyme [42].

The current study's findings regarding the potentially relevant α‐amylase inhibition efficacy of *L. strictum* fixed oil are consistent with earlier research on *Linum* species. *Linum usitatissimum* (linseed) extract showed strong α‐amylase inhibition action, including inhibition of enzymes that hydrolyze carbohydrates, according to Alawlaqi et al. (2023) [45]. This suggests that bioactive chemicals produced from flax may play a role in regulating glucose metabolism. Similarly, the particular chemical composition of *L. strictum* fixed oil, especially the presence of unsaturated fatty acids like α‐linolenic, linoleic, and oleic acids, which may contribute to enzyme modulation and metabolic regulation, may be responsible for the inhibitory effect seen against α‐amylase.

However, changes in plant species, extraction techniques, and chemical profiles may be responsible for the disparities in activity levels between linseed extract and *L. strictum* fixed oil. Although linseed extracts have a wider variety of polar phytochemicals, such as lignans and phenolic compounds, the fixed oil under investigation in this work is mostly composed of lipid content. Therefore, the combined action of *L. strictum* fixed oil's fatty acid components and small bioactive compounds may be responsible for the reported α‐amylase inhibition activity. To compare the contributions of each component and clarify the precise mechanisms involved, more research is needed.

As previously reported the abundance of bioactive chemicals in plant oil is responsible for its bioactivity [30].

According to a previous study, the strong anti‐α‐glucosidase action of several medicinal mushrooms may have been attributed to oleic and linoleic acids [46].

In fact, Wechakorn et al. (2025) found that a non‐competitive inhibition of α amylase enzyme was demonstrated by the oleic acid identified as predominant compound from crude extracts of edible insects [47].

A previous study, suggested that *satureja* fatty acids extract exhibited stronger inhibitory effects on α‐amylase (IC 50 was between 0.37 and 0.39 µg/mL) than the reference medication, acarbose [48].

Another study found that the dichloromethane extract of *Mimosa diplotricha* displayed a strong inhibitory activity against the amylase enzyme with an IC_50_ of 1792.48 µg/mL [48]. In fact, previous studies demonstrated that oleic and linoleic acids are competitive inhibitors and can bind to the active site of both amylase and glucosidase enzymes, therefore blocking starch function [46].

*L. strictum* fixed oil's ability to inhibit α‐amylase points to a possible function in slowing down the absorption of glucose and decreasing the digestion of carbohydrates. This impact could be explained by unsaturated fatty acids interacting with the active site of the enzyme to partially decrease its catalytic activity. Additionally, a molecular docking study supported the experimental results by showing favorable binding interactions between α‐amylase and the main fatty acids. Therefore, the fixed oil may be a viable natural source of chemicals with potential antidiabetic characteristics, according to the combined in vitro and in silico data [2].

The assessment was carried out solely through in vitro tests, which do not accurately replicate the intricacy of biological systems. In order to corroborate the observed hypoglycemic action as well as to assess the oil's bioavailability, safety, and pharmacological relevance, more in vivo research is required.

### 3.6 Molecular Studies

A molecular docking study determines the precise fit of the ligand molecule in the active site of the target protein, examining the mode of action of small‐molecule compounds as antimicrobial agents. The binding affinity of the ligand molecule with the target protein can be predicted by its orientation. To provide a structural rationale for the experimentally observed antibacterial activity, two well‐established bacterial therapeutic targets, β‐lactamase BEL‐1 (PDB ID: 5EUA) and DNA gyrase B (PDB ID: 4URO), were selected for a molecular docking study. These proteins were chosen because of their critical roles in bacterial survival and antibiotic resistance and because they have been extensively used as molecular targets in previous antibacterial docking studies [49, 50]. Molecular docking against these proteins was performed to evaluate whether the identified phytochemicals could theoretically interact with functionally important residues and thereby provide a plausible mechanistic explanation for the observed antibacterial effects. DNA gyrase B is an essential bacterial enzyme responsible for ATP‐dependent DNA supercoiling, a process required for DNA replication, transcription, and recombination. Consequently, inhibition of DNA gyrase disrupts bacterial DNA metabolism and has long been recognized as an effective antibacterial strategy. β‐Lactamase BEL‐1, a clavulanic acid‐inhibited Ambler class A extended‐spectrum β‐lactamase produced by *P. aeruginosa*, hydrolyzes the β‐lactam ring of penicillins, cephalosporins, and even carbapenems, thereby contributing to multidrug resistance. Since inhibition of β‐lactamase activity represents an important strategy for restoring antibiotic efficacy, BEL‐1 was selected as a biologically relevant target for evaluating the potential interactions of the identified compounds [49, 50, 51]. It should be noted that these proteins were selected because they are validated antibacterial targets relevant to the observed biological activity and are not intended to imply that they are experimentally confirmed molecular targets of the investigated phytochemicals. Therefore, the docking results provide complementary computational evidence and should be interpreted as hypothesis‐generating predictions that require further experimental validation. Definitive confirmation of ligand binding and mechanism of action requires additional biochemical and biophysical validation, such as enzyme inhibition kinetics, surface plasmon resonance, isothermal titration calorimetry, or other target‐engagement assays. This complementary strategy, in which molecular docking is integrated with experimental biological evaluation to provide mechanistic insight while acknowledging its limitations, has been increasingly adopted in recent natural product and medicinal chemistry studies [52, 53, 54, 55, 56, 57]. The two‐dimensional (2D) binding interactions of the selected ligands with the 5EUA protein are presented in Figure 4 and summarized in Table 5. Molecular docking analysis revealed that α‐oleic acid exhibited the highest binding affinity, with a docking score of −5.4 kcal/mol, followed by α‐linolenic acid (−5.0 kcal/mol), stearic acid (−4.6 kcal/mol), linoleic acid (−4.5 kcal/mol), and palmitic acid (−4.4 kcal/mol), respectively. However, the reference (standard) ligand demonstrated a substantially stronger binding affinity, with a docking score of −7.2 kcal/mol, indicating a plausible superior interaction with the target protein. Similarly, the 2D binding interactions of the ligands with the 4URO protein are depicted in Figure 5 with detailed interaction profiles provided in Table 6 Docking results for 4URO demonstrated that α‐linolenic acid showed the highest binding affinity (−5.2 kcal/mol), followed by linoleic acid (−5.0 kcal/mol), stearic acid (−4.7 kcal/mol), oleic acid (−4.6 kcal/mol), and palmitic acid (−4.5 kcal/mol). In comparison, the standard ligand exhibited the highest binding affinity toward 4URO, with a docking score of −7.7 kcal/mol, suggesting more favorable binding interactions than the investigated phytoconstituents. Molecular docking has become an important complementary tool in natural product research because it predicts ligand–protein interactions. Therefore, it has been successfully employed in various previous studies to provide mechanistic support for experimentally observed antimicrobial, anticancer, anti‐inflammatory, and enzyme inhibitory activities [58, 59, 60]. Moreover, molecular docking scores do not directly reflect inhibitory activity or establish a definitive biological mechanism. Therefore, the interactions identified in the present study should be considered hypothetical and predictive and require further experimental validation. Future studies employing direct molecular interaction assays, target‐binding studies, and enzyme inhibition assays will be necessary to confirm these predicted interactions and elucidate their potential biological relevance.

**TABLE 5:** The detailed explanation of 2D binding interactions of major compounds from fixed oil with β‐lactamase BEL‐1(PDB ID: 5EUA).

			Ligand–protein interactions			
S. No.	Ligand	Binding affinity (kcal/mol)	Alkyl Pi‐alkyl Bond	Hydrogen bond	C─H bond	van der Waals
1.	Palmitic acid (CID: 985)	−4.4	TYR A:97	SER A:62, ASN A:125	—	GLU A:159, ASN A:163, CYS A:61, SER A:230, SER A:123, GLY A:229, THR A:228, THR A:209, LEU A:122
2.	Oleic acid (CID: 445639)	−5.4	PRO A:160, TYR A:97 (Pi‐Sigma)	THR A:228, SER A:123, SER A:62	—	LYS A:227, GLY A:229, SER A:230, ARG A:236, THR A:209, GLU A:96, ASN A:125
3.	Linoleic acid (CID: 5280450)	−4.5	TYR A:97	THR A:228, SER A:230, SER A:62	—	LYS A:227, GLY A:229, SER A:123, GLU A:96, ASN A:125, LYS A:65, PRO A:160, ASN A:163
4.	α‐Linolenic acid (CID:5280934)	−5.0	TYR A:97	ASN A:163, ASN A:125, GLU A:159, SER A:62	—	THR A:213, ARG A:236, THR A:228, THR A:209, GLY A:229, SER A:230, SER A:123, CYS A:61, LYS A:65
5.	Stearic acid (CID: 5281)	−4.6	TYR A:97, PRO A:160	SER A:62, ASN A:125, SER A:230, ASN A:163	—	GLU A:159, LYS A:65, GLY A:229, CYS A:61, SER A:123, GLU A:96, CYS A:231
6.	^a^Ciprofloxacin (CID: 2764)	−7.2	—	PRO A:160; ASN A:163; ASN A:125; SER A:123; LYS A:65	SER A:230; THR A:228	CYS A:231; LYS A:227; GLY A:229; THR A:209; SER A:62; TYR A:97; GLU A:159

^a^ = Standard.

FIGURE 52D binding interaction of ligands in the active site of the DNA gyrase protein (PDB: 4URO: (a) Palmitic Acid‐4URO; (b) Oleic Acid‐4URO; (c) Linoleic Acid‐4URO; (d) α‐Linolenic Acid‐4URO; (e) Stearic Acid‐4URO; (f) Ciprofloxacin‐4URO.

**Figure:**
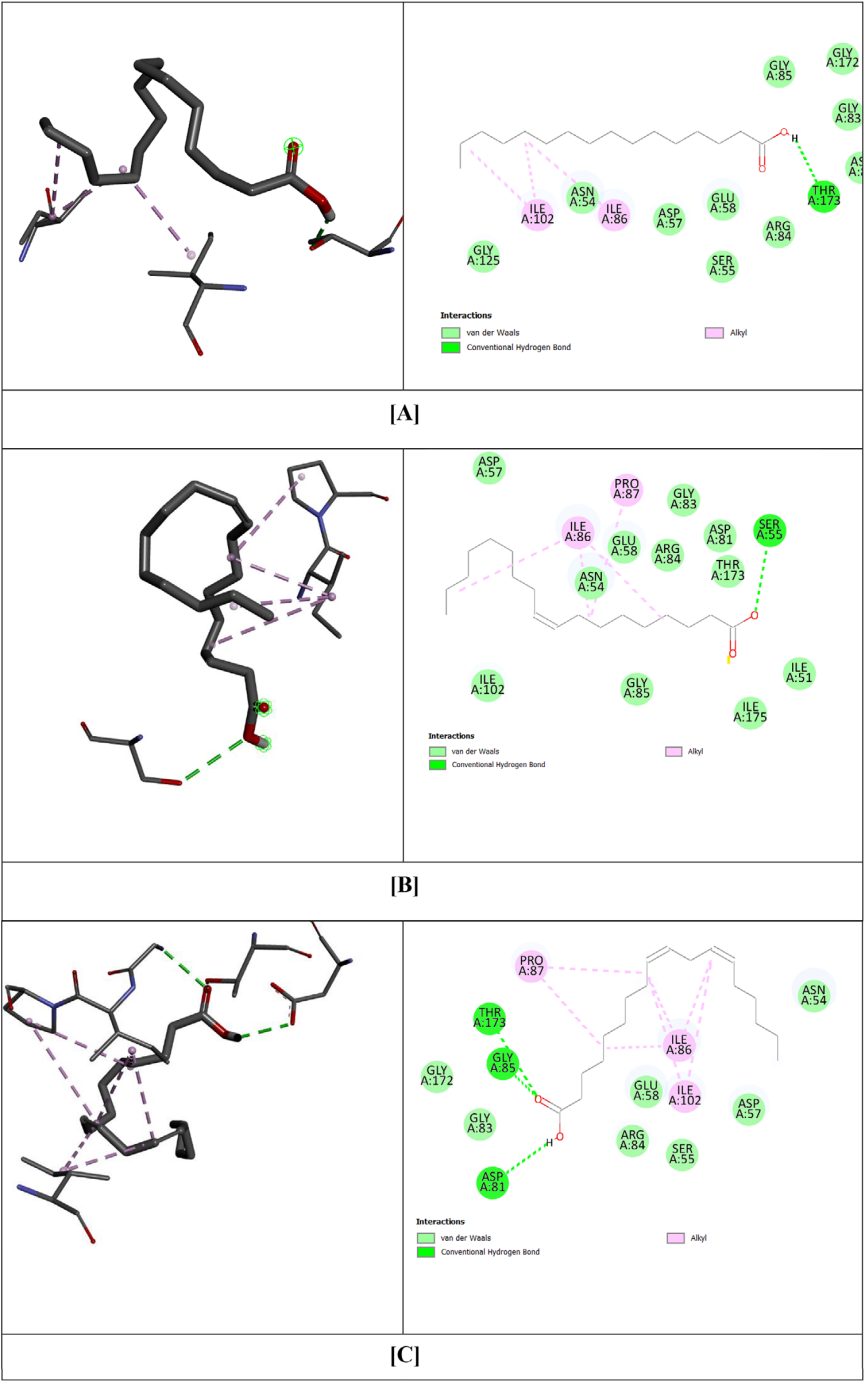

**Figure:**
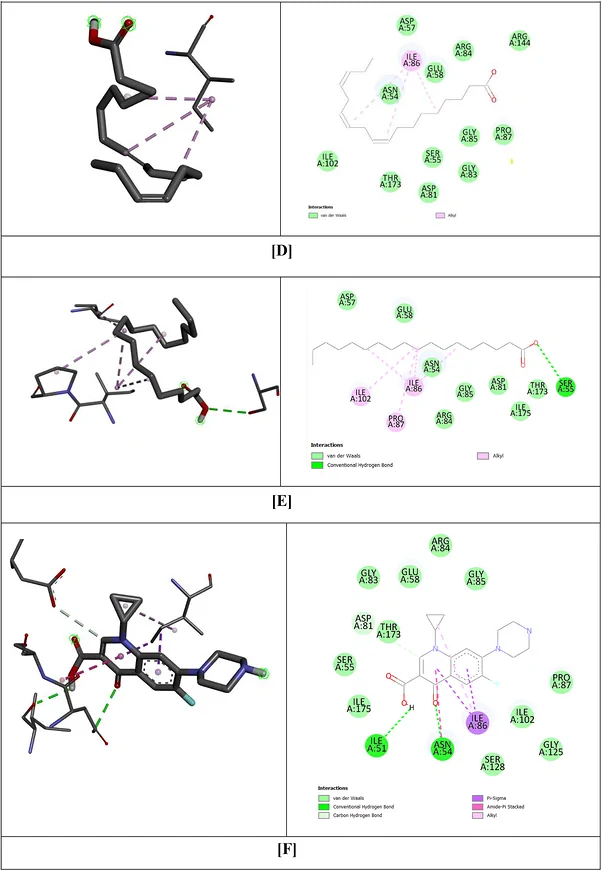

**TABLE 6:** The detailed explanation for 2D binding interactions of major compounds from fixed oil with DNA gyrase B (PDB ID: 4URO).

			Ligand–protein interactions			
S. No.	Ligand	Binding Affinity (‐kcal/mol)	Alkyl and Pi‐sigma Bond	C─H bond	Hydrogen bond	van der Waals
1.	Palmitic acid (CID: 985)	−4.5	ILE A:102, ILE A:86	—	THR A:173	GLY A:85, GLY A:172, GLY A:83, ASP A:81, ARG A:84, GLU A:58, SER A:55, ASP A:57, ASN A:54, GLY A:125
2.	Oleic acid (CID: 445639)	−4.6	ILE A:86, PRO A:87,	—	SER A:55	ASP A:57, ILE A:102, ASN A:54, GLY A:85, GLU A:58, ARG A:84, GLY A:83, ASP A:81, THR A:173, ILE A:51, ILE A:175
3.	Linoleic acid (CID: 5280450)	−5.0	ILE A:86, ILE A:102, PRO A:87	—	THR A:173, GLY A:85, ASP A:81	GLY A:172, GLY A:83, GLU A:58, ARG A:84, SER A:55, ASP A:57, ASN A:54
4.	α‐Linolenic Acid (CID:5280934)	−5.2	ILE A:86	—	—	ASP A:57, ASN A:54, ILE A:102, THR A:173, ASP A:81, SER A:55, GLY A:83, GLY A:85, PRO A:87, ARG A:144, ARG A:84, GLU A:58
5.	Stearic acid (CID: 5281)	−4.7	ILE A:86, PRO A:87, ILE A:102	—	SER A:55	ASP A:57, GLU A:58, ASN A:54, ARG A:84, GLY A:85, ASP A:81, ILE A:75, THR A:173
6.	^a^Ciprofloxacin (CID: 2764)	−7.7	ILE A:86	ASP A:81	ASN A:54; ILE A:51	GLY A:85; ARG A:84; GLU A:58; GLY A:83; THR A:173; SER A:55; ILE A:175; SER A:128; ILE A:102; GLY A:125; PRO A:87

^a^ = Standard.

It should be noted that while the molecular docking analysis was carried out in our study, utilizing specific individual fatty acid constituents found in the oil, the antibacterial and antibiofilm actions discovered in the current work were experimentally assessed using the entire fixed oil. Therefore, it is not possible to directly interpret the expected interactions of oleic acid, α‐linolenic acid, and other individual fatty acids with bacterial protein targets as proof that each of these molecules is responsible for the biological activity of the entire oil. It is impossible to rule out the possibility of synergistic, additive, or antagonistic interactions between the various components of the fixed oil that may have contributed to the reported activity. Therefore, rather than directly explaining the experimentally reported antibacterial or antibiofilm effects of natural compounds, the docking results should be viewed as preliminary computational evidence of possible molecular interactions [53, 54]. To determine their individual contributions and potential interactions, more research utilizing defined combinations of the primary ingredients and isolated fatty acids, together with suitable target‐specific assays, would be required.

## 4 Conclusions

To conclude, *L. strictum* seed oil was rich in unsaturated fatty acids (89.75%), including linoleic acid (14%), oleic acid (17.53%), and alpha‐linolenic acid (57.08%). An interesting antibacterial effect was observed against *Enterococcus faecalis ATCC* 29212*, A. baumannii* A722, and *A. baumannii* A2553 with MIC = 1.25 mg/mL. In addition, more than 70% of *A. baumanniii* that formed biofilm was inhibited at a concentration of 2XMIC. Significant anticoagulant activity on the intrinsic pathway by the prolongation of KTC coagulation time. Finally, when compared to acarbose, the positive control, which showed 84.93% inhibition and an IC_50_ of 0.015 mg/mL, our results showed an interesting inhibitory potential of *L*. *strictum* seed oil, exhibiting 85.98% inhibition with an IC_50_ value of 0.0134 mg/mL.

Our findings suggested that *L*. *strictum* seed oil could be considered a potent antibiofilm agent against multidrug‐resistant bacteria such as *A. baumannii*, as well as having interesting anticoagulant and α‐amylase inhibition effects.

Preliminary in silico evidence of possible interactions between specific fatty acid contents and bacterial protein targets was provided by the docking analysis. Before any conclusive mechanism of antibacterial action can be established, these hypothesized interactions should be regarded as speculative and require experimental validation.

This integrated approach presents novel insights into the pharmacological potential of *L. strictum* fixed oil and broadens the current knowledge regarding its possible biomedical applications. In vivo validation is required to determine whether these findings can be translated into safe and effective antimicrobial–adjuvant strategies.

## Author Contributions

**Hayet Edziri** designed the experimental work and wrote the article. **Pooja Bargali** and **Ravendra Kumar** performed the docking study. **Marwa Melliti** participated in the experimental part of antimicrobial activity. **Hasan Aljohi** and **Mohammad NasserAlkhrayef** data/samples collection; they contributed to chemical analysis. **Rim Gtari** data analysis. **Marcello Iriti** supervision and revised the manuscript. **Ibrahim O Alanazi** contributed by chemicals and English revision,

## Funding

The authors have nothing to report.

## Conflicts of Interest

The authors declare no conflicts of interest.

## Data Availability

All data generated or analyzed during this study are included in this published article
